# Immobilization of Horseradish Peroxidase on NH_2_-Modified Magnetic Fe_3_O_4_/SiO_2_ Particles and Its Application in Removal of 2,4-Dichlorophenol

**DOI:** 10.3390/molecules191015768

**Published:** 2014-09-29

**Authors:** Qing Chang, Heqing Tang

**Affiliations:** Key Laboratory of Catalysis and Materials Science of the State Ethnic Affairs Commission and Ministry of Education, College of Chemistry and Materials Science, South-Central University for Nationalities, Wuhan 430074, China; E-Mail: changqinghust@163.com

**Keywords:** magnetite nanoparticles, silica, immobilized enzyme, horseradish peroxidase, degradation, 2,4-dichlorophenol

## Abstract

Fe_3_O_4_ nanoparticles were prepared by a co-precipitation method with the assistance of ultrasound irradiation, and then coated with silica generated by hydrolysis and condensation of tetraethoxysilane. The silica-coated Fe_3_O_4_ nanoparticles were further modified with 3-aminopropyltriethoxysilane, resulting in anchoring of primary amine groups on the surface of the particles. Horseradish peroxidase (HRP) was then immobilized on the magnetic core-shell particles by using glutaraldehyde as a crosslinking agent. Immobilization conditions were optimized to obtain the highest relative activity of the immobilized enzyme. It was found the durability of the immobilized enzyme to heating and pH variation were improved in comparison with free HRP. The apparent Michaelis constants of the immobilized HRP and free HRP with substrate were compared, showing that the enzyme activity of the immobilized HRP was close to that of free HRP. The HRP immobilized particles, as an enzyme catalyst, were used to activate H_2_O_2_ for degrading 2,4-dichlorophenol. The rapid degradation of 2,4-dichlorophenol indicated that the immobilized enzyme has potential applications for removing organic pollutants.

## 1. Introduction

Horseradish peroxidase (HRP), an enzyme isolated from the roots of horseradishes, is the most widely used catalyst in enzymatic reactions [1,2]. Recently, it is found that in the presence of hydrogen peroxide [3] HRP can be used to remove chlorophenols, which have been labeled as “priority pollutants” by the US Environmental Protection Agency, from contaminated water [4]. However, HRP is not stable under various conditions. The stabilization of HRP may be achieved by medium engineering, chemical crosslinking, protein engineering or enzyme immobilization [5,6].

Enzyme immobilization can endow enzymes with some additional advantageous properties. The immobilized enzymes can be used repeatedly or continuously in a variety of reactors for the efficient recovery of costly enzymes, and be easily separated from reaction systems for reuse, which make the work-up simple and the protein of the final product uncontaminated [7,8,9]. Furthermore, it is reported that immobilized enzymes may exhibit higher selectivity and specificity [10]. Nevertheless, during the immobilization procedure, enzymes may be denatured and lose their activity because of distortions, especially when multi-interactions between the enzyme and the support occur [6]. Besides, the immobilization may block the active center of the enzyme and lead to diffusion problems. These two problems may have a different impact on the activity of the enzyme depending on the different immobilization strategies [10]. In principle, enzymes are immobilized via three major routes: (i) binding to a support; (ii) encapsulation or entrapment; or (iii) cross-linking. Immobilization by covalent attachment to water-insoluble carriers via glutaraldehyde is one of the simplest and most gentle coupling methods in enzyme technology because the reaction proceeds in aqueous buffer solution under conditions close to physiological pH, ionic strength, and temperature. Essentially, two methods of the formation of a three-dimensional network as a result of intermolecular crosslinking and the binding to an insoluble carrier have been used [11]. Many chemists have tried to increase the performance of immobilized enzymes, such as specific activity, storage, and recycling stability, and ease of reuse. Recent breakthroughs in nano and hybrid technology have made various materials more affordable hosts for enzyme immobilization [12].

Nanoparticles are considered to be an ideal support for enzyme immobilization due to their minimized diffusional limitations, maximum surface area per unit mass, and high enzyme loading capability [12,13,14]. However, when using non-porous nanoparticle supports, the purity of the enzyme sample only impairs the volumetric activity [10]. Additionally, it is necessary to find a more effective way for separating and recycling the nanoparticles [15]. As a magnetic material, iron oxide nanoparticles have been widely used in many fields such as separation of biochemical products [16], magnetically assisted drug delivery [17] and enzyme immobilization [18]. Iron oxide nanoparticles can be modified with functional groups or inorganic compounds to obtain a magnetic carrier promising for enzyme immobilization [19]. Silica particles are hydrophilic, biocompatible and stable in most biosystems. Moreover, silica chemistry is well-known, and standard chemistry protocols can be followed to conjugate various biomolecules to the silica surface [20], which thus turns out to be a very good solid support for enzyme immobilization [21].

In the present work, the NH_2_-modified magnetic silica particles were prepared and HRP were immobilized on the surface of the particles. Immobilization conditions were optimized on the relative activity of the immobilized enzyme. Finally, the HRP-immobilized magnetic silica particles were applied to remove 2,4-dichlorophenol in the presence of H_2_O_2_.

## 2. Results and Discussion

### 2.1. Synthesis of HRP-immobilized Magnetic Particles

**Scheme 1:**
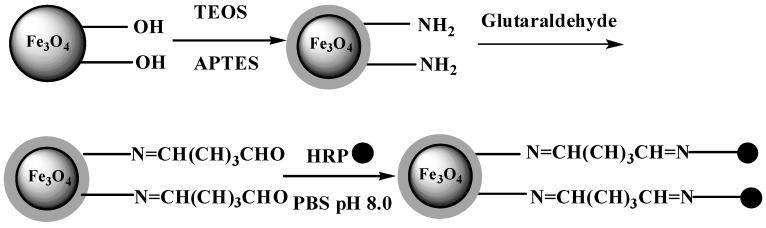
Synthesis of NH_2_-modified Fe_3_O_4_/SiO_2_ particles.

The procedures for the synthesis of NH_2_-modified Fe_3_O_4_/SiO_2_ particles and the immobilization of HRP are illustrated in Scheme 1. Firstly, magnetic nanoparticles were prepared via ultrasound-assisted reverse co-precipitation as in our previous report [22]. In Wang’s report, the XPS spectrum indicated that the products are Fe_3_O_4_. There are slight differences between the XRD patterns of magnetite and maghemite, although their patterns are similar. Weak peaks at 15.0°, 18.4°, 23.8° and 26.1°, corresponding the diffraction of (110), (111), (210) and (210) of maghemite (PDF 39–1346), could not been seen. However, only one weak peak at 18.2° was present in XRD pattern in Wang’s report. Therefore, the product prepared by ultrasound-assisted reverse co-precipitation is indeed magnetite and the method was thus adopted to synthesize magnetite in the present work. Additionally, the synthesized iron oxide is black, a typical color of magnetite, not the brownish red color of maghemite, which also confirmed that the iron oxide product we prepared is magnetite. Then the magnetite nanoparticles were coated with silica generated by the hydrolysis and condensation of tetraethoxysilane (TEOS). Subsequently, the NH_2_-modified magnetic silica particles were obtained by adding 3-aminopropyltriethoxysilane (APTES). Finally, HRP was immobilized on the surface of the silica particles by using glutaraldehyde (GA) as a covalent crosslinker.

### 2.2. Characterization

**Figure 1:**
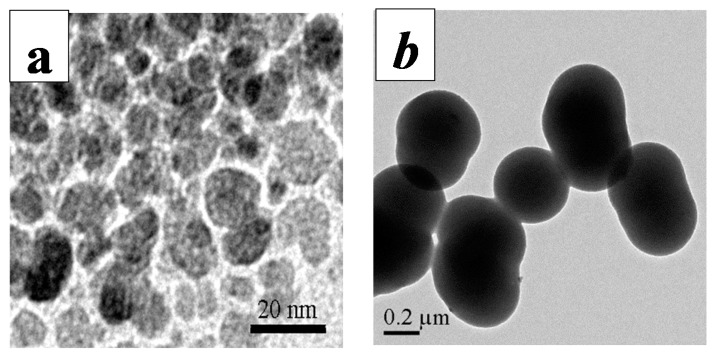
TEM images of Fe_3_O_4_ (**a**) and NH_2_-modified Fe_3_O_4_/SiO_2_ particles (**b**).

The TEM images of Fe_3_O_4_ and NH_2_-modified Fe_3_O_4_/SiO_2_ particles are given in Figure 1. Figure 1a indicates that the nanoparticles were spherical, with diameters ranging from 10 to 15 nm. For NH_2_-modified Fe_3_O_4_/SiO_2_, it is evident that the particles were spherical in shape and exhibited a smooth surface, and some of them were found to aggregate together (Figure 1b). The NH_2_-modified Fe_3_O_4_/SiO_2_ particles had a core diameter of about 10 nm and a silica-shell thickness of 195 nm on average, yielding an average total diameter of 400 nm. The thickness of the silica shell can be adjusted from a few to several hundreds of nanometers by simply varying the initial amount of TEOS [23].

**Figure 2:**
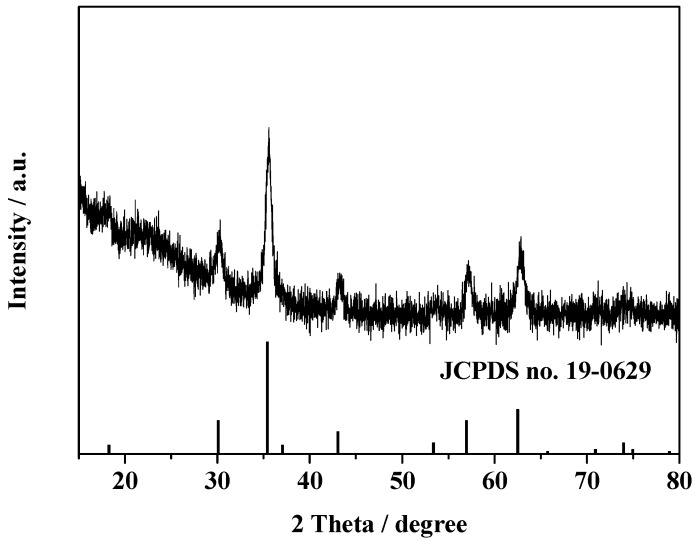
XRD pattern of NH_2_-modified Fe_3_O_4_/SiO_2_ particles.

The crystalline structure of NH_2_-modified Fe_3_O_4_/SiO_2_ particles was investigated by XRD. As shown in Figure 2, the diffraction peaks (2θ) at 18.2°, 30.3°, 35.6°, 43.3°, 53.8°, 57.3° and 62.8° are ascribed to the (111), (220), (311), (400), (422), (511) and (440) planes of Fe_3_O_4_, respectively, which coincide well with the standard data of Fe_3_O_4_ (JCPDS no. 19-0629).

**Figure 3:**
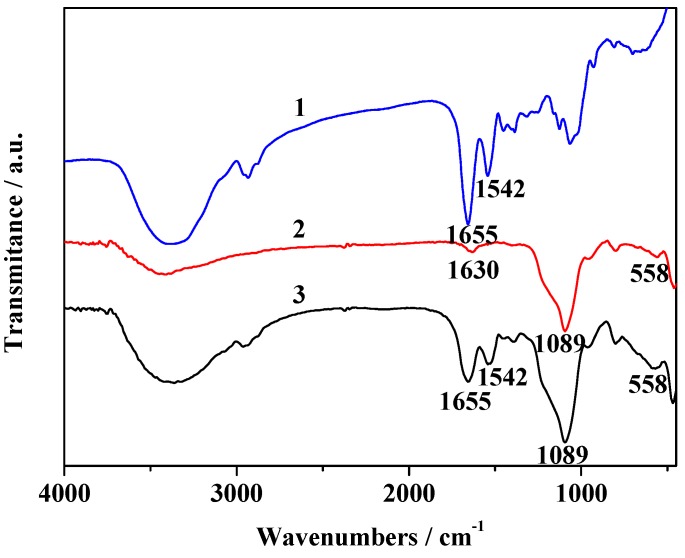
FTIR spectra of HRP (1), NH_2_-modified Fe_3_O_4_/SiO_2_ (2) and immobilized HRP (3).

Figure 3 shows the FTIR spectra of NH_2_-modified Fe_3_O_4_/SiO_2_ particles, immobilized HRP and free HRP, respectively. The characteristic absorption of Fe-O vibration is observed at 558 cm^−1^ (curve 2). In the case of NH_2_-modified Fe_3_O_4_/SiO_2_ particles (curve 2) the sharp band at 1089 cm^−1^ corresponds to Si-O-Si antisymmetric stretching vibrations, being indicative of the existence of SiO_2_ in the particles. The characteristic absorption band for N-H bond was observed at 1630 cm^−1^. For HRP, the characteristic adsorptions at 1655 and 1542 cm^−1^ are attributed to -CONH- (amide I) and amide II vibrations, respectively. Immobilized HRP also displays these bands, indicating that HRP has been successfully immobilized on the NH_2_-modified Fe_3_O_4_/SiO_2_ particles.

### 2.3. Effect of Concentration of GA

**Figure 4:**
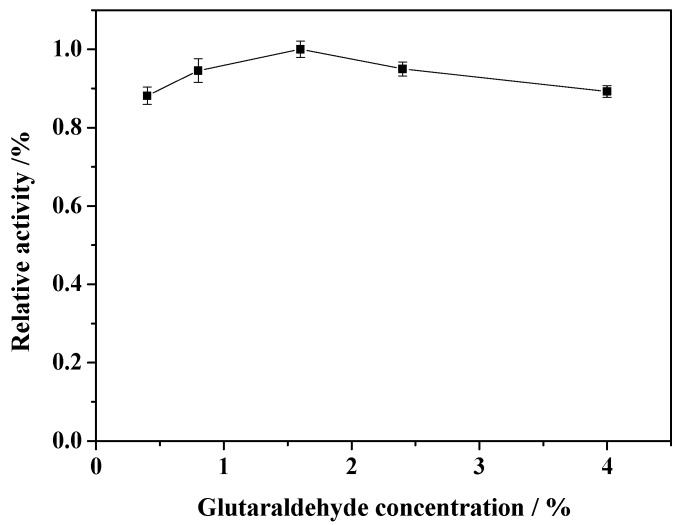
Effect of GA concentration on the relative activity of the immobilized enzyme.

The concentrations of GA must be carefully considered to obtain water-insoluble enzyme derivatives via crosslinking because low concentrations of enzyme and GA tend to induce intramolecular crosslinking by enhancing the probability that GA functional groups will react with the same enzyme molecule. The effect of the concentration of GA (in the immobilization process) on the activity of immobilized enzyme was shown in Figure 4. 

When the concentration of GA is less than 1.6%, immobilized HRP activity is increased with increasing concentration of GA, and then the activity declines when the GA concentration is higher than 1.6%. This is due to the fact that low concentrations of GA are not able to form sufficient crosslinkages to effect precipitation of the enzyme, and at higher concentrations, the extent of crosslinking is high enough to form a tight structure by excluding water molecules to insolubilize the enzyme derivative [11]. In addition, it is indicated that extensive crosslinking may result in a distortion of the enzyme structure. With this distortion, the accessibility and accommodation of the substrate may be reduced, thus affecting the retention of enzyme activity [24]. Thus, the concentration of GA was optimized at 1.6% in these experiments.

### 2.4. Effect of Immobilization Time

**Figure 5:**
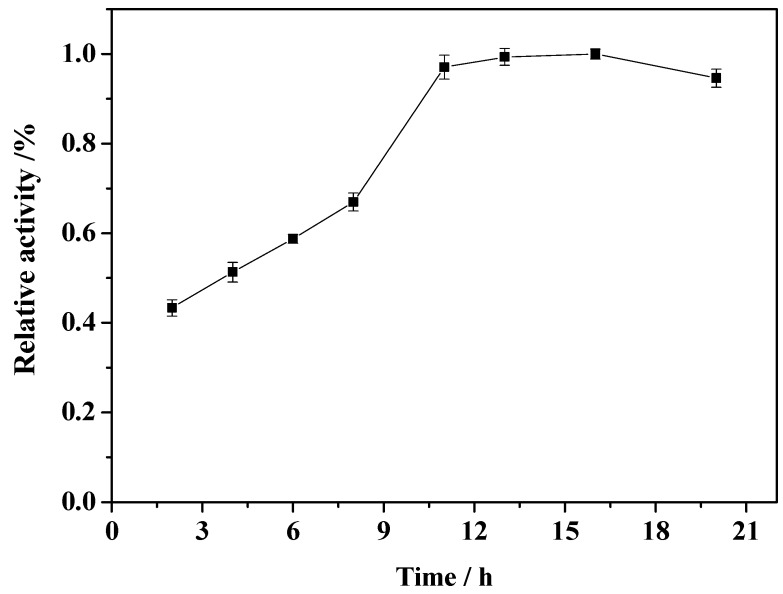
Effect of immobilization time on the relative activity of the immobilized enzyme.

The effect of immobilization time on the relative activity of the immobilized enzyme was investigated. As shown in Figure 5, the activity of the immobilized enzyme initially increases rapidly and then essentially reaches a plateau beyond 11 h. This is because the immobilization of enzyme on the carrier is saturated after 11 h. With prolonged reaction time, any increase in the amount of immobilized enzyme may increase significantly the steric hindrance to the immobilized enzyme molecules, leading to slightly decreased enzyme activity. According to this observation, the reaction time was optimized at 11 h.

### 2.5. Effect of Reaction Temperature

**Figure 6:**
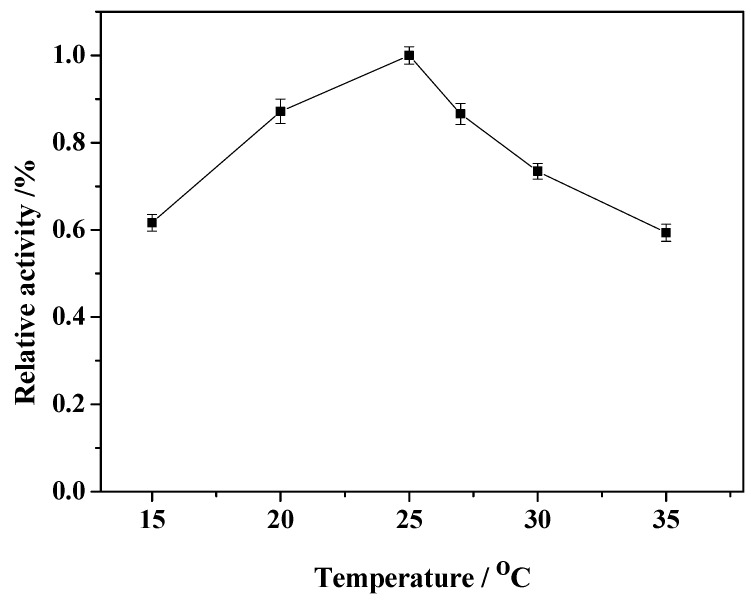
Effect of immobilization temperature on the relative activity of the immobilized enzyme.

During the immobilization reaction, on the one hand, the crosslinking reaction between enzymes, the crosslinking agent and the carrier should be carried out effectively; on the other hand, the enzyme must maintain its activity during the immobilization. Thus, the proper reaction temperature is very important. To examine the effect of immobilization temperature on the relative activity of the immobilized enzyme, the reaction was performed at temperatures ranging from 15 to 35 °C (Figure 6). At low temperature region (25 °C and lower), the activity of immobilized enzyme increases with the rise of temperature, yielding a activity maximum at 25 °C. As the temperature is further increased, the immobilized enzyme activity decreased because of inactivation of the enzyme at high temperature. Consequently, the reaction temperature was optimized at 25 °C in this experiment.

### 2.6. Effect of pH

**Figure 7:**
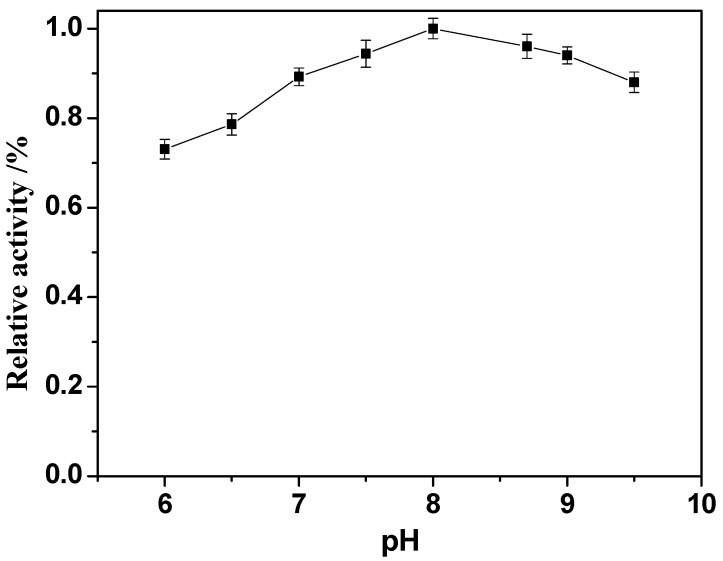
Effect of immobilization pH on the relative activity of the immobilized enzyme.

In the immobilization process, the solution pH can change the dissociation status of the enzyme and free state of the carrier, and vary the spatial conformation of the enzyme, thus affecting its binding capacity, resulting in differences in the relative activity of the enzyme. Therefore, the influence of solution pH on the immobilized enzyme reaction was studied. As shown in Figure 7, the reaction with amino groups proceeds better with the increase of pH, and the pH value for optimum activity was found to be pH 8.0. The relative activity of the immobilized enzyme is reduced when pH > 8.0 because of the inactivation of enzyme. Therefore, the reaction solution pH was optimized at pH 8.0 for the immobilization.

### 2.7. Stability of the Immobilized Enzyme

**Figure 8:**
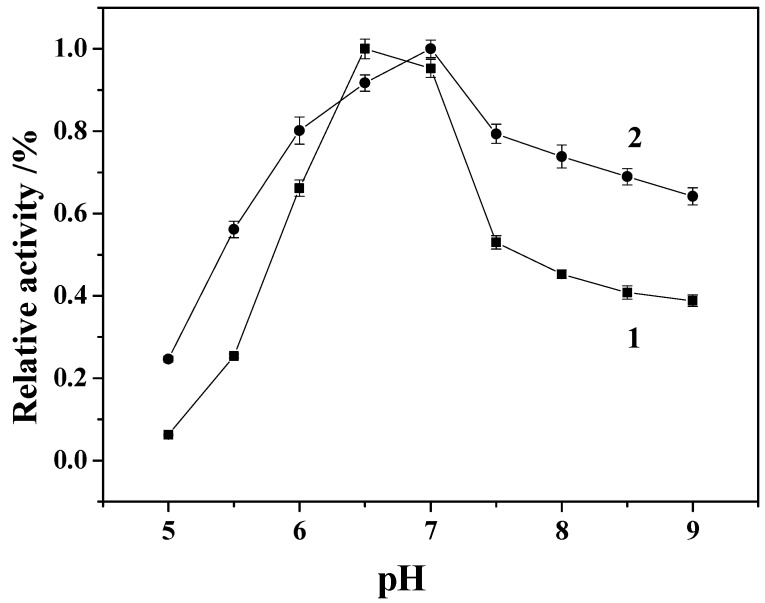
Effect of pH on the activity of the free enzyme (1) and immobilized enzyme (2).

The immobilized enzyme should have good stability during its use in the oxidative removal of organic pollutants. Thus the effect of solution pH on the activity of both the free and immobilized enzyme in the pH range from 5.0 to 9.0 was investigated as shown in Figure 8. The optimum pH values for the free HRP and immobilized HRP were found to be at pH 6.5 and 7.0, respectively. The reason is that the carrier can absorb H^+^ from the reactive solution to its surface, causing lower pH in the surrounding of the immobilized enzyme than that of bulk reactive solution. Thus, the pH of this area should be raised to a certain amount so as to enable the enzyme to function properly. As a result, the pH of the immobilized enzyme appears to be higher than that of free enzymes [25]. The relative activity of free and the immobilized enzyme is reduced when the pH is too acid or too alkaline. The above results suggest that the immobilized enzyme is more resistant to acid and alkaline conditions than the free HRP.

**Figure 9:**
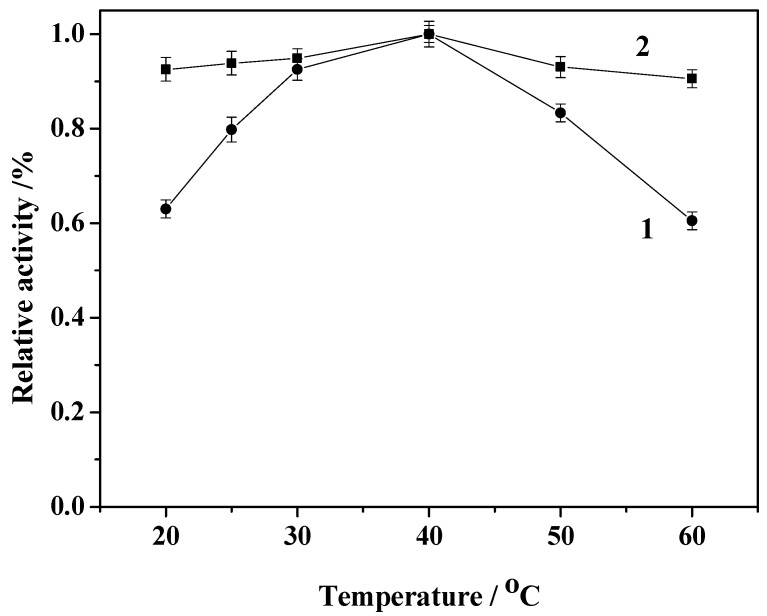
Effect of temperature on the activity of the free enzyme (1) and immobilized enzyme (2).

Figure 9 shows the effect of temperature on the relative activity of free HRP and immobilized HRP. It can be seen that the relative activity of free HRP is increased with the increase of temperature in the range from 20 to 40 °C, and it started to decrease with increasing temperature beyond 40 °C. The immobilized HRP also has maximal activity at 40 °C, but the activity of the immobilized enzyme is less sensitive to temperature than that of the free enzyme. Enzymes can be immobilized via ionic exchange, hydrophobic interactions, or covalent attachment with the assistance of the versatile crosslinker glutaraldehyde. As the reaction between amino/glutaraldehyde moieties will occur in a wide range of pH values, primary amino group-modified Fe_3_O_4_@SiO_2_ support molecules may react with HRP besides the intramolecular and intermolecular crosslinking of HRP enzyme by the glutaraldehyde crosslinker during its immobilization. Thus the support will behave as a large multi-crosslinking reagent, fixing the positions of all the enzyme groups involved in the reaction with the support, promoting an increase of the overall enzyme structure and thus protecting HRP from inappropriate changes at high temperatures [26,27]. The immobilized enzyme thus endures a much wider range of temperatures.

### 2.8. Kinetic Parameters

The enzyme kinetic constant K_m_ (Michaelis constant) is one of the most important factors for the evaluation of an enzyme’s efficiency in order to understand the enzymatic characteristics of an immobilized enzyme. For an enzyme-catalyzed process, the reaction rate (ν) and the concentration of the substrate ([*c*]) will obey the Michaelis-Menten equation, in which the basic parameters can be determined from the Lineweaver-Burk plots according to Equation (1) [28]:

(1) $$\frac{1}{\nu }=\left(\frac{K_{m}^{app}}{\nu_{\max}}\right)\frac{1}{\left[c\right]}+\frac{1}{\nu_{\max}}$$

**Figure 10:**
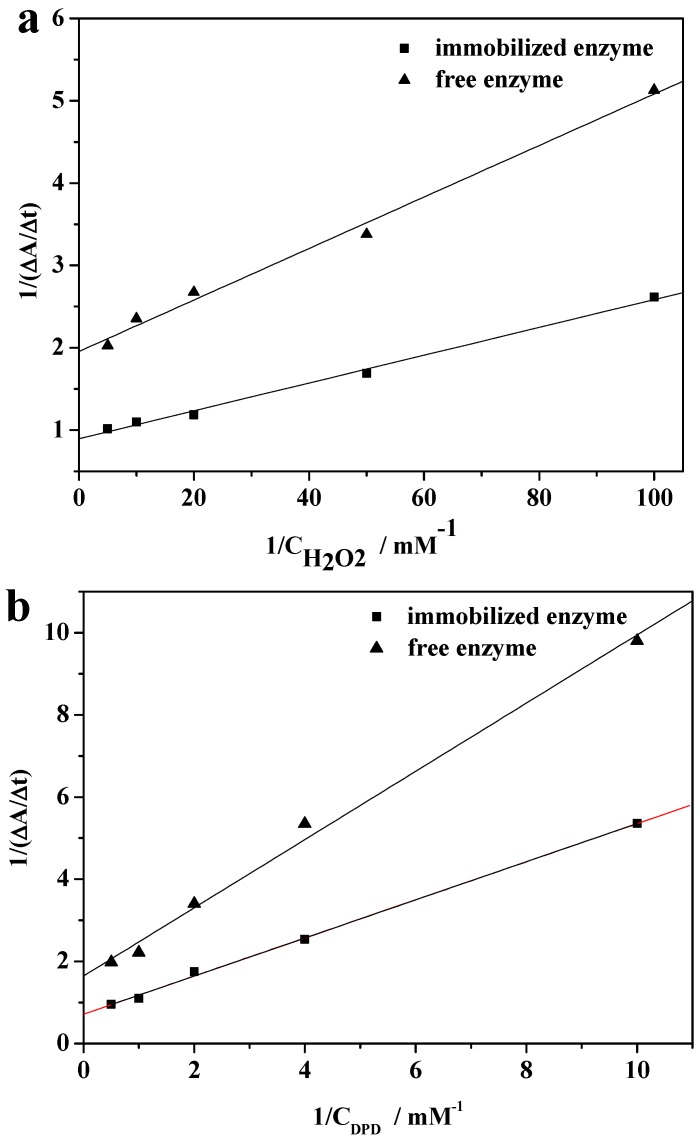
Lineweaver-Burk plots of the free enzyme and immobilized enzyme with H_2_O_2_ (**a**) and DPD (**b**) as the substrate.

The data were fitted to the Michaelis-Menten model to obtain the related parameters. Figure 10a shows that the apparent K_m_ value of the immobilized HRP with H_2_O_2_ as the substrate (0.019 mM) is only slightly greater than that of free HRP (0.016 mM), which indicates that the affinity of the immobilized enzyme for the substrate is close to that of the free HRP for the substrate. As shown in Figure 10b, the apparent K_m_ value of the immobilized HRP (0.65 mM) with *N*,*N*-diethyl-*p*-phenylenediaminesulfate (DPD) as the substrate is also only slightly greater than that of HRP (0.50 mM), suggesting that the immobilized HRP also has an equally high affinity as free HRP. The slightly decreased affinity of the immobilized enzyme for the substrate in comparison with that of the free enzyme is because of the increased steric hindrance. However, due to the enzyme-like activity of Fe_3_O_4_ nanoparticles [29], such weakening is not as strong and the affinity of the immobilized HRP for the substrate is still high.

### 2.9. Catalytic Degradation of 2,4-Dichlorophenol

**Figure 11:**
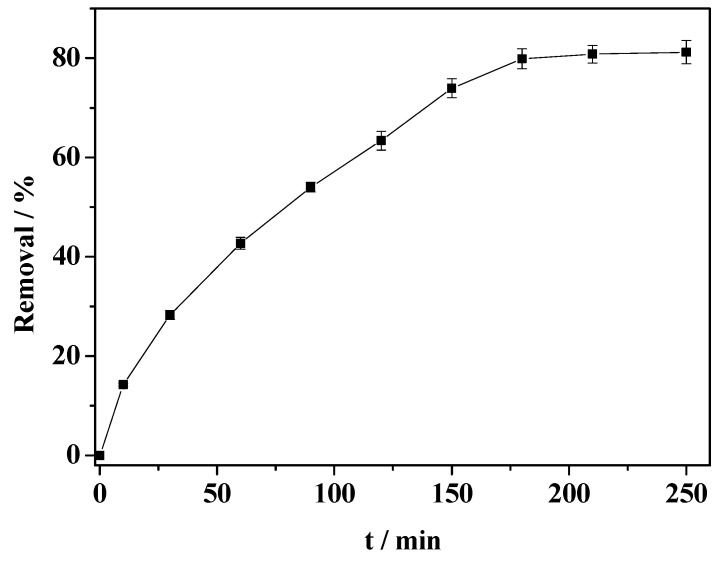
Time profiles of the removal of 2,4-dichlorophenol.

The HRP-immobilized magnetic particles were used as an enzyme catalyst to activate H_2_O_2_ for degrading 2,4-dichlorophenol. As a co-substrate, H_2_O_2_ activates the enzymatic reaction to produce peroxidase radical intermediate, which attacks chlorophenol compounds to form a free radical [30]. The optimization for the removal of chlorophenol was performed by measuring the removal by changing the concentration of H_2_O_2_ but fixing the concentration of 2, 4-dichlorophenol at 0.2 mmol·L^−1^. The optimum molar ratio of H_2_O_2_ to 2,4-dichlorophenol in this experiment was 1.0. Figure 11 shows the degradation of 2,4-dichlorophenol with the use of the immobilized HRP in the presence of H_2_O_2_ at pH 6.4 and 30 °C. As shown in Figure 11, the removal of 2,4-dichlorophenol initially increases rapidly and then reaches a platform beyond 180 min, with a maximum removal of about 80%. The results indicate that the immobilized enzyme has potential for the removal of organic pollutants from water.

### 2.10. Reusability

**Figure 12:**
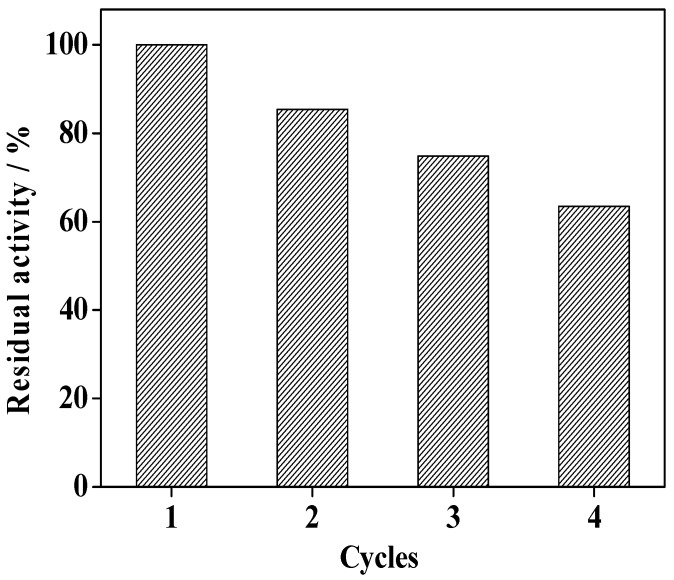
Reusability of the immobilized enzyme in 2,4-dichlorophenol removal.

After the used catalysts were recovered and washed with water to remove the substrate and products, the reusability of the immobilized enzyme was investigated. As shown in Figure 12, the immobilized enzyme retains about 85% of its initial activity after four cycles, indicating that the immobilized HRP has appropriate stability and can be reused.

## 3. Experimental Section

### 3.1. Reagents

HRP was purchased from Tianyuan Biologic Engineering Corp. (Wuhan, China). TEOS and APTES (99%) was obtained from Aldrich (Shanghai, China). GA (25% w/v aqueous solution) was purchased from the Shanghai Chemical Reagent (Shanghai, China). All other chemicals were of analytical grade and were used as received. The water was double distilled water. 

### 3.2. Preparation of NH_2_-Modified Fe_3_O_4_/SiO_2_ Particles

Fe_3_O_4_ magnetic nanoparticles were synthesized by ultrasound-assisted reverse co-precipitation as reported previously [22]. Fe_3_O_4_ nanoparticles (2.0 g) were added to a mixture of ethanol (200 mL) and distilled water (40 mL) and sonicated for 10 min. Under continuous stirring, ammonia solution (30 wt %, 5.0 mL) and TEOS (6.0 mL) were added into the above suspension, and the reaction was allowed to continue for 3 h under stirring. Then APTES (2.0 mL) was added, and the mixture was agitated for 10 h at room temperature. After the reaction was completed, the products were collected through centrifugation, followed by washing with water and ethanol several times, and then dried overnight in an oven to afford core-shell magnetic particles.

### 3.3. HRP Immobilization

PBS buffer (pH 7) containing magnetic particles carriers was mixed with 1.6% GA at 25 °C for 3 h. Then the carriers were washed with the buffer solution and distilled water. The GA-treated particles (1.6 g) and HRP (2 mg) were dispersed in a PBS buffer (pH 8.0). The mixture was incubated with a shaker at 150 rpm for 11 h at 25 °C. The particles were collected by magnetic separation and washed with phosphate buffer and water to remove non-specifically bound enzyme. The enzyme-immobilized particles were redispersed in water and stored at 4 °C. A colorimetric procedure was used to determine the enzyme activity [31].

### 3.4. Catalytic Experiments

Immobilized enzyme solution (1.0 mL) was dispersed into aqueous solution of 2,4-dichlorophenol (40 mL, 0.2 mmol·L^−1^) at pH 6.4. The reaction mixture was vibrated at a speed of 200 rpm at 25 °C. After 30 min to achieve adsorption-desorption equilibrium, the reaction was initiated with the addition of H_2_O_2_ (0.2 mmol·L^−1^). At given time intervals, 2 mL aliquots of the reaction solution were sampled, and immediately recovered by magnetic separation. The concentration of 2,4-dichlorophenol remaining in solution was determined by a spectrophotometric method [32].

### 3.5. Characterization

As-synthesized products were observed with a FEI Tecnai G2 20 transmission electron microscope (FEI, Burlington, VT, USA). Powder X-ray diffraction pattern was collected on a Bruker Advance D8 X-ray powder diffractometer (Bruker AXF, Karlsruhe, Germany), with Cu KR radiation (40 kV, 40 mA). The FTIR spectra were recorded in the 400–4000 cm^−1^ range on a Nicolet Nexus 470 FTIR spectrometer (Thermo Nicolet, Waltham, MA, USA).

## 4. Conclusions

Magnetite nanoparticles were synthesized by an ultrasound-assisted co-precipitation method, and then silica-coated magnetic particles were prepared through the Stöber method. The silica-coated Fe_3_O_4_ particles were treated with APTES in order to obtain NH_2_-modified magnetic particles and HRP was further covalently immobilized on the composites by using GA as a coupling agent. Batch optimization studies were carried out to demonstrate the effects of initial solution pH, GA concentration, temperature, and time of the immobilization process on the relative activity of the immobilized enzyme. It was found that the immobilized enzyme was more resistant to heating and pH variation than free HRP. The immobilized enzyme could be used as an enzyme catalyst for the enzymatic degradation of 2,4-dichlorophenol, thus proving to be a potential efficient catalyst for treatment of organic pollutants.
